# The Influence of Conventional or KOBO Extrusion Process on the Properties of AZ91 (MgAl9Zn1) Alloy

**DOI:** 10.3390/ma14216543

**Published:** 2021-11-01

**Authors:** Piotr Długosz, Włodzimierz Bochniak, Paweł Ostachowski, Rafał Molak, Martin Duarte Guigou, Marek Hebda

**Affiliations:** 1 Centre of Casting Technology, Research Network Lukasiewicz-Krakow Institute of Technology, Zakopiańska 73, 30-418 Kraków, Poland; 2 Faculty of Non-Ferrous Metals, AGH University of Science and Technology, A. Mickiewicza 30 Av., 30-059 Kraków, Poland; bow@agh.edu.pl (W.B.); pawel.ostachowski@agh.edu.pl (P.O.); 3 Faculty of Materials Science, Warsaw University of Technology, Wołoska 141, 02-507 Warszawa, Poland; rafal.molak@pw.edu.pl; 4 Faculty of Mechanical Engineering, Bialystok University of Technology, Wiejska 45c, 15-351 Białystok, Poland; 5 Department of Engineering and Technology, Catholic University of Uruguay Damaso Antonio Larrañaga, Av. 8 de Octubre 2738, Montevideo 11600, Uruguay; martinduarte79@gmail.com; 6 Faculty of Material Engineering and Physics, Cracow University of Technology, Warszawska 24, 31-155 Kraków, Poland

**Keywords:** magnesium alloys, KOBO method, superplasticity, microstructure, mechanical properties

## Abstract

Designers’ efforts to use the lightest possible materials with very good mechanical properties mean that in recent years magnesium alloys have been increasingly used. It is well-known that the use of various plastic working processes allows achieving even better strength properties of the material, often without significant loss of plastic properties in relation to the properties of products obtained in the casting process. The article presents the results of research on microstructural changes and mechanical properties of the alloy AZ91 (MgAl9Zn1) occurring in samples subjected to conventional plastic deformation and the KOBO method. The obtained results were compared to the properties of reference samples, i.e., cast samples. The article presents the advantage of using the low-temperature KOBO method compared to the high-temperature deformation in a conventional manner. Moreover, it has been shown that the use of KOBO extrusion allows the alloy AZ91 (MgAl9Zn1) to obtain superplasticity properties with an elongation of up to 577% compared to the cast reference sample, which is generally classified as difficult for plastic deformation.

## 1. Introduction

In recent years, the use of magnesium alloys as construction materials has become more and more popular. This is a consequence of the relatively easy access to the natural resources of this element, as well as the popular methods of its processing and recycling. Magnesium is the eighth-most abundant element in the Earth’s crust, at 2.74%. However, it acquires the appropriate strength properties only in the form of alloys, which are commonly considered the lightest construction material. The deformability of magnesium alloys is limited by the structure of their crystallographic lattice. The dominant plastic deformation mechanisms include dislocation slip, twinning, and grain boundary slip. The phenomenon of dynamic recrystallization can also be observed [1]. As magnesium crystallizes in the A3 hexagonal pattern, alloys based on magnesium can be processed mainly at elevated temperatures. Plastically processed products made of magnesium alloys are characterized by higher strength and plastic properties compared to products obtained in the casting process. Among the alloys intended for plastic processing, there is no AZ91 (MgAl9Zn1) alloy, which due to its good strength at room temperature, excellent castability, dimensional stability, sea corrosion resistance, average aluminum content of about 9% by weight, is considered to be a typical foundry alloy. On the other hand, alloys from the Mg-Al-Zn system, with Al content from 1% to 8% by weight, are recommended for plastic processing. Due to its good mechanical properties and good technological characteristics, AZ91 (MgAl9Zn1) alloy is very often used for lightweight cast structural parts. It is widely used, among others, for the production of castings for the automotive industry, mainly by pressure casting methods, and to a lesser extent it is cast into permanent molds (molds), as well as for disposable molds (sand, gypsum, and ceramic) [2,3,4]. Despite the fact that AZ91 (MgAl9Zn1) alloy is included in the group of casting alloys, various forms of its plastic forming are not excluded. Reguła et al. [5] presented results of laboratory tests of hot extrusion of AZ91 (MgAl9Zn1) alloy in which the properties of extruded products and castings processed according to different variants of heat treatment were compared. Rajendran et al. [6] in their work focused on the analysis of the parameters of the AZ91 (MgAl9Zn1) alloy extrusion process. Tan et al. [7] investigated the structural changes of AZ91 (MgAl9Zn1) alloy squeezed at 380 °C. On the other hand, Thirumurugan et al. [8] analyzed the microstructural changes of the AZ91 (MgAl9Zn1) alloy extruded at the following temperatures: 250 °C, 300 °C, 350 °C. Kim et al. [9] found that the so-called extrudability and mechanical properties of AZ91 (MgAl9Zn1) alloy can be adjusted by introducing the additional factor—water cooling, during extrusion. Currently, the most advanced research related to the processing of magnesium alloys on an industrial scale relates to extrusion processes. The so-called extrudability of magnesium alloys is measured, inter alia, by the extrusion rate. Magnesium alloys are extruded at a slower speed than aluminum alloys, but with greater force. Too high squeezing speed favors the formation of blisters, hot brittleness, and a reduction in mechanical properties. On the other hand, reducing the extrusion speed may not be economical. The extrusion processes include the production of bars and sections as well as profiles of various cross-sections. Currently, the best results have been obtained for the processes of co-extrusion of magnesium pipes with the use of mandrels, while as for the other methods of plastic working, the progress in the field of their forging and stamping is clearly noticeable. At the same time, innovative methods of rolling sheets of magnesium alloys [10,11,12,13,14,15] are being developed. For example, Kurz et al. [10] presented the results of the casting experiments with the use of twin rolls of AZ31 (MgAl3Zn1) magnesium alloy tapes. Bergea et al. [11] showed results of the influence of temperature on the mechanical properties of the twin-roll cast, rolled, and heat-treated AZ31 (MgAl3Zn1), investigated under tensile loading from different directions. Hu et al. [12] analyzed the development of a model of the twin-roll casting of magnesium alloy by uniting equations of temperature and flow fields. Results of the experimental rolling process with AZ31 (MgAl3Zn1) in extended temperatures have been presented in [13]. Neh et al. [14] focused on opportunities for the production of strips via twin-roll casting and strip rolling of several magnesium alloys containing aluminum or rare earth elements. Whereas, Minoa et al. [15] investigated the properties of the wrought AZ61 magnesium alloy after twin-roll process. A number of SPD (Severe Plastic Deformation) methods [16,17,18,19,20,21,22,23,24,25], and methods of superplastic deformation [26,27] are currently under development. Among the methods of SPD, it is enough to mention: multi pass tubular channel angular pressing (TCAP) [16], large strain rolling [17], accumulative roll bonding (ARB) [18], accumulative back extrusion (ABE) [19], cyclic extrusion compression (CEC) [20], equal-channel angular pressing (ECAP) [21,22,23], and high pressure torsion HPT [24,25].

An economical and efficient method of deformation, which creates a chance to obtain a stable and fragmented microstructure of the product, is the cold extrusion of metal through a die twisted on both sides, the so-called KOBO method. The method was developed at the AGH University of Science and Technology as a structural criterion for designing plastic processing processes of metallic materials [28,29]. It consists of taking complete control of the evolution of the substructure and the related mechanical properties of the deformed metal through external interference in the manner of its plastic flow. In particular, being the essence of the KOBO method, properly induced periodic changes in the deformation path lead to a several-fold reduction of the plastic resistance, which in practice means not only a significant reduction of the process energy, but above all obtaining the desired, fine-grained and homogeneous structure, and thus high functional properties [30]. Technological processes based on the KOBO method, in relation to conventional processes, are enriched with a factor that causes cyclical changes in the deformation path in the deformed material as a result of cyclical changes in its load pattern [31,32,33]. According to the patent claims, this factor is usually a mechanism which is a source of additional torque, operating in a predetermined plane in a cyclical manner and with a defined frequency. In the case of the KOBO method, this factor causes permanent destabilization of the metal structure with the domination of localized plastic flow in cyclically intersecting shear bands. According to the appropriate procedure accompanying the application of the KOBO method to plastic working processes, it is possible to carry out “cold” (i.e., below the recrystallization conditions of a given material) plastic forming processes with a speed and degree of processing much higher than in high-temperature processes, and the product obtains a fine-grained, homogeneous structure and favorable mechanical properties [30]. Moreover, maintaining a constant value of the extrusion force by adjusting the oscillation frequency of the die and the speed of the punch movement is conducive to obtaining the same material properties along the entire length of the sample [34,35]. The KOBO method was used during research on plastic deformation of hard-to-deform materials, including: MMC (Metal Matrix Composite), aluminum alloys (7075), titanium, bronze (CuSn8), magnesium alloys (AZ31 (MgAl3Zn1), AZ91 (MgAl9Zn1), AZ61 (MgAl6Zn1)) [36,37,38], and compared to the results obtained for typical plastic forming processes, such as extrusion, pressing, forging, rolling or drawing [39,40]. The KOBO technology was also used for the direct production of wire (omitting the liquid phase) from aluminum scrap (e.g., from aluminum cans), waste shavings from aluminum and titanium alloys [41,42], and waste from magnesium alloys.

It is well-known that metals and their alloys with a finely divided structure, even up to the nanometric scale, have superplastic properties [43,44]. The reduction in grain size should lead to a reduction in the optimal temperature for the occurrence of superplasticity or to an increase in the optimal strain rate at which this phenomenon occurs. This assumption underlies the design of most SPD methods. Research results obtained and described by the teams of Mukherije [44] and Valiev [45,46,47] had a considerable influence on the course of research on the mechanisms of superplastic flow. According to Mukherijee, superplasticity is a property of polycrystalline materials that tend to achieve enormous elongations before tensile fracture. In other words, superplasticity defines this phenomenon as the ability of materials to undergo great plastic deformation without disturbing internal cohesion, appearing at high homologous temperatures under the influence of stresses, the magnitude of which is extremely low and strongly dependent on the rate of deformation. As a result of stretching, there is resistance to the formation of the neck or the so-called multi-neck and the tendency to huge, even exceeding 1000%, elongations. According to the theory of Mukherjee [48,49,50], the superplastic flow of microcrystalline materials is often described by the equation determining the dependence of the strain rate on the stress.

Superplastic properties were revealed, inter alia, during the tests of the PbSn60 alloy [51,52] of the Inconel 718 alloy [53], the aluminum alloy A1MgSi [54] and in magnesium alloys, including AZ91 (MgAl9Zn1) [55,56,57,58,59]. Matsubara et al. [55] examined cast AZ91 (MgAl9Zn1) alloy after conventional hot extrusion and through angular channel extrusion. He observed a reduction in grain size from 50 μm in the cast state to 12 μm after conventional extrusion and ~0.7 μm after conventional extrusion and then angular extrusion at 200 °C. It was found that as a result of the combination of conventional extrusion and extrusion through an angular channel, the alloy showing moderate plasticity in the as-cast state obtained superplastic properties—800% elongation at 150 °C. On the other hand, the work [56] showed that AZ 91 alloy with an average grain size of 4 μm after the Friction Stir Processing (FSP) process allowed to obtain an elongation of the material at the temperature of 330 °C at the level of 1251% and 827%. In [57], the process of extrusion of the conventional AZ91 (MgAl9Zn1) alloy at 250 °C was carried out, followed by angular extrusion at 175 °C. In the static tensile test performed at the temperature of 200 °C with the speed of 6 × 10^−5^ s^−1^, the elongation of 661% was obtained. Wei et al. [56] achieved 455% elongation in rolled AZ91 (MgAl9Zn1) magnesium alloy at high strain rates. Al-Zubaydi et al. [25] presented the results of research on the superplasticity of samples made of AZ91 (MgAl9Zn1) alloy, conventionally extruded and additionally deformed by the HPT (High Pressure Torsion) method at room temperature. The analyzed samples obtained even 760%, 1164% and 1308% elongation. The use of superplastic properties of magnesium alloys allows for the production of very complex and at the same time light and durable details with thin walls and of any shape [58].

The article presents the results of research on microstructural changes and mechanical properties occurring in samples subjected to conventional extrusion and extrusion by the KOBO method. The obtained results were compared to the properties of reference samples, i.e., cast samples. Moreover, in order to verify whether the cast magnesium alloy AZ91 (MgAl9Zn1) subjected to plastic deformation could exhibit superplastic properties, tensile tests were carried out at the temperature of 300 °C and 350 °C.

## 2. Methodology and Materials

The alloy AZ91 (MgAl9Zn1) was used for the tests. The composition was determined on the basis of chemical analysis carried out with the GDS-850A LECO optical emission spectrometer (LECO, 3000 Lakeview Ave. St. Joseph, MI, USA). The obtained results were compared with the values from the PN-EN 1753: 2020-01 standard [59] and presented in Table 1.

AZ91 (MgAl9Zn1) alloy ingots with dimensions Ø 40 × 50 mm for the KOBO process and Ø 96 × 100 mm for extrusion process, were produced by casting into a disposable sand mold. A molding material in the form of a mixture of sand and SUPER-ECO resin was used to make the sand mold. AZ91 (MgAl9Zn1) alloy was melted in a specialized resistance furnace (PTM-15/G, CZYLOK, Jastrzębie Zdrój, Poland) in a steel crucible in a protective SF6 atmosphere. The FLUX preparation was used for the refining of the alloy. The preparation protected the bath against ignition. Metal melting took place at a temperature of 680–700 °C. The molten metal was poured into the sand mold. After the metal solidified, the mold was broken, and the casting was machined.

The extrusion process was carried out at a temperature of 370 °C using a horizontal counter-rotating hydraulic press (ZAMET, Tarnowskie Góry, Poland) with a stamp force of 500 T. The extrusion speed was 0.5 mm/s. In this way, a press iron with a diameter of 15 mm and λ = 45 was produced.

**Figure 1:**
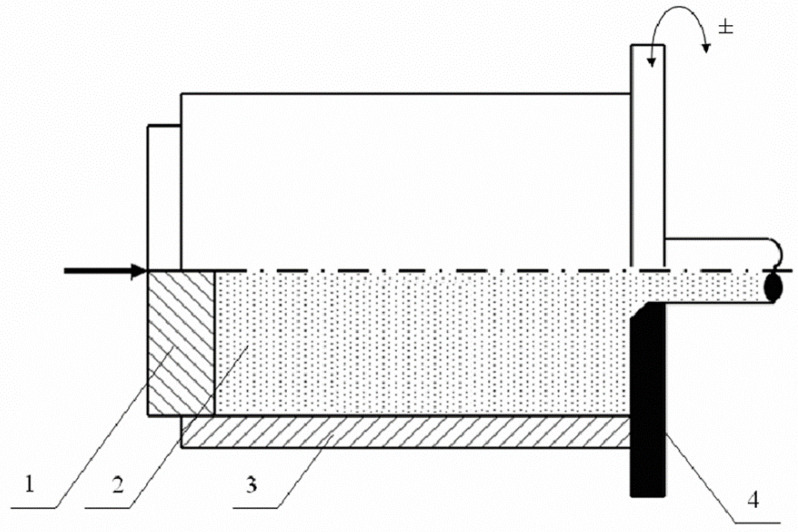
Scheme of the extrusion process using the KOBO method: (1) stamp, (2) ingot, (3) container, (4) reverse twisted die.

Extrusion with the KOBO method was carried out at ambient temperature with the use of the KOBO laboratory stand for extruding metals and alloys through a die rotated on both sides (Figure 1).

The stand consists of a horizontal hydraulic press (Hydromet, Bytom, Poland) with a punch force of 100 T equipped with a module that allows for double-sided (reverse) rotation of the die, a recipient and a cooled die holder. The rotation angle of the die was 8° and the frequency was 3 or 6 Hz. The alloy was deformed to a diameter of Ø 6 and λ = 44.4. The extrusion speed was 0.1 mm/s.

Metallographic examinations were performed on the Zeiss Axio Observer Z1m light microscope (Carl Zeiss, Jena, Germany). The metallographic specimens were etched with a 4% solution of HNO_3_ in ethyl alcohol. Microscopic observations were also carried out using the SCIOS-FEG, FEI scanning electron microscope, equipped with a chemical composition microanalyzer.

Static tensile tests for cast materials, after extrusion and the KOBO process, were carried out on samples with a measuring base length of L_0_ = 25 mm and a measuring diameter of d_0_ = 5 mm in accordance with the PN-EN ISO 6892-1:2020-05 [60]. The samples were stretched at ambient temperature at a speed of 8 × 10^−4^ s^−1^.

Superplasticity tests were carried out on samples with a measuring base length equal to L_0_ = 30 mm and a measuring diameter of d_0_ = 4 mm, the total length of the sample was Lt = 50 mm, and the thread length (M6) was 9 mm.

The coefficient m, called the stress sensitivity to the strain rate, whose high value characterizes the superplastic state, was determined on the basis of the formula:

(1) $$m=\frac{\partial \log\sigma }{\partial \log\dot{\varepsilon }}$$

where: σ—stress $\dot{\varepsilon }$—strain rate.

## 3. Results and Discussion

**Figure 2:**
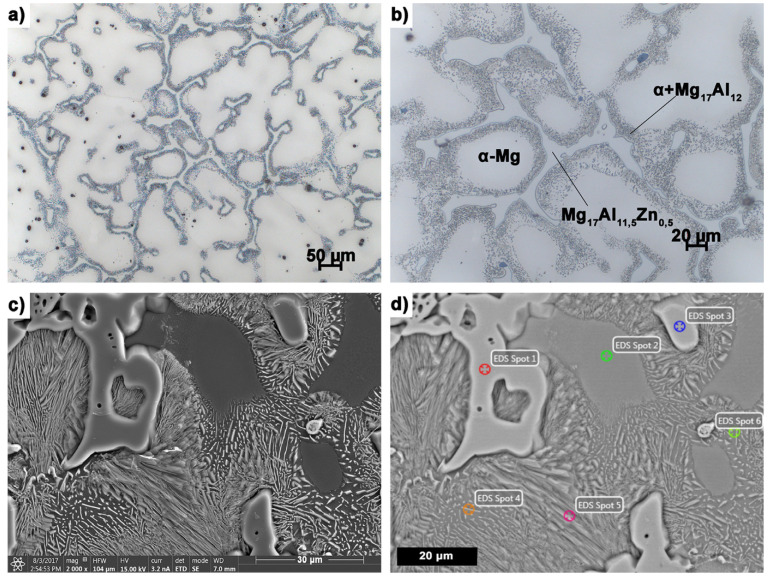
Microstructure of AZ91 (MgAl9Zn1) sand casting magnesium alloy recorded with (**a**,**b**) optical microscope, (**c**,**d**) SEM.

The primary microstructure of the sand casting is shown in Figure 2. Crystallization of this type of casting is slower than in the case of castings cast into metal molds due to the heat balance in the liquid alloy-mold material system.

It is heterogeneous, with a clear dendritic microsegregation of the alloy components. This is the effect of an unstable crystallization front during the solidification of the sand casting. It causes the components with lower solubility in the α-Mg solution to move further from the crystallization front.

The matrix of the alloy is a solid solution of α-Mg, which is confirmed by the results of microanalysis in microarea 2 (Figure 2d). The microstructure shows the discontinuous precipitates of Mg_17_Al_12_ phases with lamellar morphology (Figure 2b–d) and massive precipitations of the pre-eutectic phase, which, based on the results of the microanalysis of the chemical composition (Figure 2, Table 2) and the presence in them Zn atoms substituted for the Al subnetwork (micro-area 1 in Figure 2d) can be identified as Mg_17_Al_11.5_Zn_0.5_, or Mg_17_(Al,Zn)_12_ (Figure 2b–d) [61]. Moreover, the multi-component eutectic of Mg + Mg_17_Al_12_ can be distinguished in the microstructure (Figure 2, micro-area 5 in Figure 2d). It is a characteristic component of the microstructure of the AZ91 (MgAl9Zn1) alloy casting, and its morphology depends on both the alloy composition (including Al concentration) and the solidification rate. The lamellar morphology of the Mg_17_Al_12_ phase precipitates with a clear growth anisotropy may be caused by the directional correlation of crystallographic orientations with the α phase [62,63,64].

**Figure 3:**
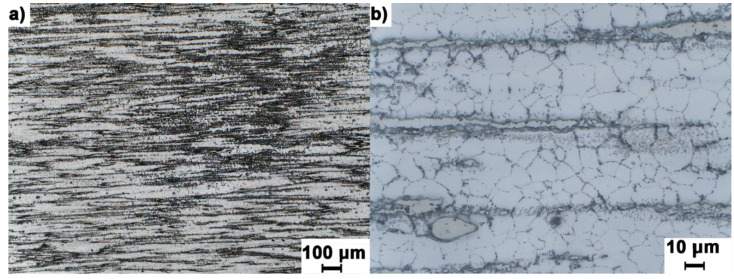
Microstructure of sand casting AZ91 (MgAl9Zn1) magnesium alloy after extrusion with the conventional method with the degree of processing λ = 45, longitudinal section observed with different magnification, (**a**,**b**) respectively.

Figure 3 shows the characteristic distinct fibrous in the direction of extrusion the microstructure of the sand casting of AZ91 (MgAl9Zn1) alloy conventionally extruded with a processing degree of λ = 45. At higher magnifications (Figure 3b) the presence of regular and equiaxed grains between the strongly elongated strands was observed. This effect is the result of dynamic recrystallization occurring during deformation at elevated temperature.

**Figure 4:**
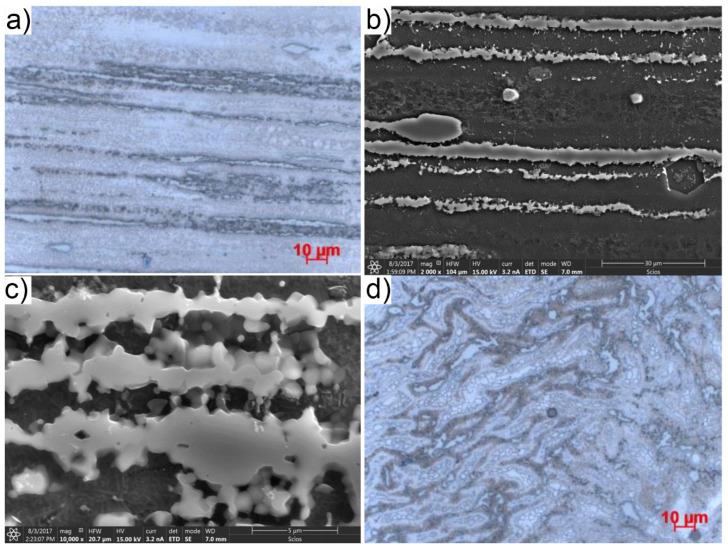
Microstructure of AZ91 (MgAl9Zn1) sand casting magnesium alloy after KOBO extrusion with the processing degree λ = 44.4 and the die rotation frequency f = 6 Hz (**a**–**c**) longitudinal section, (**d**) cross section.

Figure 4 shows a representative microstructure of microstructure of sand casting extruded by the KOBO method with a processing degree of λ = 44.4.

Similarly to the extrusion process with the conventional method, as a result of plastic deformation carried out under the conditions of the KOBO extrusion process, the primary microstructure has changed into a band, one closely related to the extrusion direction (ED). The anisotropy of the microstructure applies to both the solid solution, the biphasic eutectic and the pre-eutectic phase (Figure 4). The matrix of the microstructure is constituted by the α-Mg solution, while the phase of the Mg_17_Al_12_ type, as a result of plastic deformation, is fragmented and globularized and is situated in bands. On the other hand, the swirls of flow characteristic only for the KOBO method are observed for the cross-section of the flow turbulence (Figure 4d).

The average grain size of the conventionally extruded sand casting microstructure and the KOBO method, measured by the secant method, while maintaining a similar degree of λ processing, is 13.46 and 2.08 μm, respectively. On the basis of the obtained results, it can be unequivocally stated that the KOBO method affects the grain refinement more than six times more intensively than the conventional method of extrusion.

Table 3 presents a summary of the strength and plastic properties of the sand casting from AZ91 (MgAl9Zn1) magnesium alloy and after it was subjected to the extrusion process using the conventional and KOBO methods.

**Figure 5:**
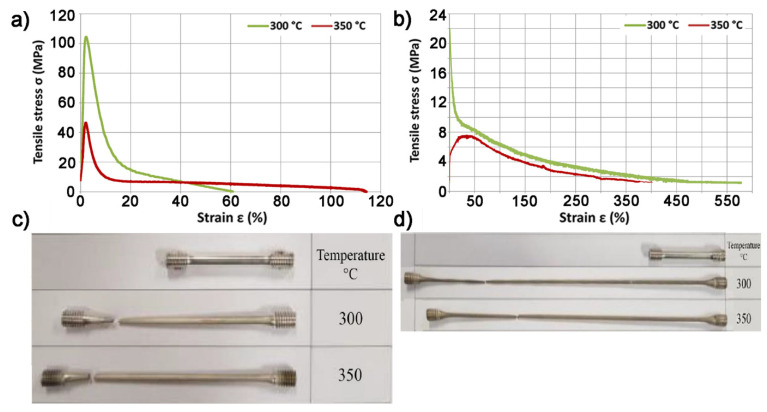
Representative tensile curves at 300 °C and 350 °C magnesium sand casting AZ91 (MgAl9Zn1) extruded using (**a**) the conventional method with the processing degree λ = 45 and ε = 10^−4^ s^−1^ (**b**) the KOBO method with the processing degree λ = 44.4 and the die rotation frequency f = 6 Hz. A photo after a static tensile test of representative samples extruded using the method (**c**) conventional, (**d**) KOBO.

Based on the data contained in Table 2, it was found that the AZ91 (MgAl9Zn1) magnesium alloy cast, made in a disposable sand mold, was characterized by a strength Rm of 125 MPa and an elongation of 1.6%. The use of the conventional extrusion process carried out at the temperature of 370 °C allowed for more than a two and a half times increase in strength properties and more than thirteen times increase in plastic properties (elongation) of AZ91 (MgAl9Zn1) magnesium alloy. In the case of sand casting extruded using the KOBO method with the processing degree of λ = 44.4, the measurements were carried out for two die rotation frequencies, f = 3 Hz and f = 6 Hz. Additionally, test samples were taken from the initial, middle and final sections of the press. The study of strength properties of conventionally extruded bars was not carried out for samples taken from different sections of the press, because the conventional process does not generate dynamic changes in the structure of the material as a function of the deformation path. A significant dependence on changes in strength and plastic properties was observed depending on the section of the AZ91 (MgAl9Zn1) magnesium alloy sample subjected to the analysis. The samples analyzed from the initial section of the press showed the highest strength properties and the lowest plastic ones. Along with the material extrusion process using the KOBO method, its strength properties decreased (from 437 to 330 MPa) and plastic properties increased (from 1.8% to 8.8%). The observed effect is typical for the extrusion process using the KOBO method with a constant frequency of the die rotation, during which two opposing mechanisms occur [65]. In the initial stage of the process, there is a relatively low supersaturation of the alloy with point defects, which takes place to an increasing extent during the process. This process leads to an increase in the strength properties of the extruded material. However, with the duration of the process, the temperature of the extruded alloy increases as a result of internal friction and friction in the tool-material system. This leads to the phenomenon of dynamic recrystallization opposing the material strengthening process. As a result, it increases the plasticity of the extruded alloy, and the appearance of new recrystallized grains in the microstructure can be observed. Therefore, regardless of the applied die rotation frequency (f = 3 and 6 Hz), the highest strength and the lowest plastic properties were recorded for samples taken from the initial section of the sample. On the other hand, the use of a lower die rotation frequency, f = 3 Hz, allowed strengthening of the strength properties of the alloy, while the die rotation frequency of 6 Hz significantly increased the plasticity properties of the alloy. The change in the direction of the die rotation, leading to a change in the deformation path, is accompanied by an intensive process of dislocation intersection and the generation of point defects (vacancies and interstitial atoms), which is a consequence of dragging dislocation thresholds and the formation of dipoles. As in the case of intersecting dislocations, their greatest concentration occurs in the outer layer of the twisted metal. Excessive concentration of point defects in relation to the equilibrium concentration and their gradient on the radius of the cyclically twisted material are the dominant elements of the plastic flow mechanism activated in the KOBO extrusion process [66]. The increase in the die rotation frequency influences the intensification of the processes generating shear stress, the increase of the process temperature and, consequently, the grain refinement. The conventionally and KOBO extruded samples were deformed in a static tensile test at 300 °C and 350 °C (Figure 5).

On the basis of the obtained results, it was found that the stretching of the conventionally extruded magnesium alloy at the temperature of 300 °C with the speed of ε = 10^−4^ s^−1^ allowed the sample deformation to increase up to 61%. This is an over 250 times better result compared to the same material obtained but deformed at ambient temperature. An increase of 50 °C in the temperature at which the tensile test was carried out, from 300 °C to 350 °C, resulted in an almost twofold increase in the material deformation to the value of 114%.

Regardless of the stretching temperature used, much higher strain values were recorded for the samples extruded using the KOBO method compared to the conventional method. When the process was carried out at the temperature of 300 °C with the speed ε = 10^−4^ s^−1^, the processing degree λ = 44.4 and the die rotation frequency f = 6 Hz, the greatest elongation of the alloy was noted, amounting to 577%. An increase in the test temperature by 50 °C decreased the plastic properties of the sample and its maximum deformation before breaking was 406%.

**Figure 6:**
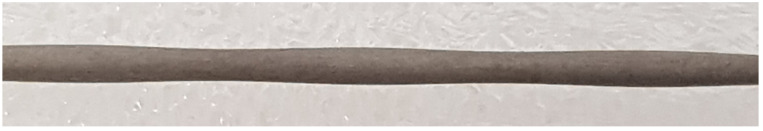
Macroscopic multi-neck effect observed for the sand casting of AZ91 (MgAl9Zn1) magnesium alloy extruded by the KOBO method with the processing degree of λ = 44.4 and the die rotation frequency f = 6 Hz stretched at 300 °C with the speed ε = 10^−4^ s^−1^.

The presented results prove that under the extrusion conditions using the KOBO method, the AZ91 (MgAl9Zn1) foundry alloy obtained superplastic properties in the high-temperature tensile test. In addition, it is also evidenced by the macroscopic effect of the so-called “multi-neck” (Figure 6), the appearance of which is usually a manifestation of the resistance to the flow of the alloy to neck formation. It consists of a temporary increase in the deformation resistance of the neck in response to the increase in the deformation velocity caused by the location of the deformation under the conditions of simultaneous reduction of the deformation zone. During hot deformation, the processes of deformation hardening and softening of the material related to the processes of structure renewal work simultaneously. If the strengthening process in the formed neck is so large that plastic deformation cannot be realized, the neck will be created in another place not yet strengthened to such an extent.

On the basis of the recorded data, the value of the sensitivity coefficient for the deformation velocity “m” was determined. It has been experimentally confirmed that if the value of “m” increases, the elongation before failure also increases. When the relationship between σ and $\dot{\varepsilon }$ is linear (“m” = 1), the material can be classified as perfectly superplastic, with extreme elongation. In the case of superplastic metals, the “m” values usually do not exceed 0.8. Most elemental metals and metal alloys have an “m” factor well below 0.1–0.2. On the other hand, alloys strengthened by dispersion, e.g., with oxides, are characterized by the coefficient “m” of about 0.02.

The value of the strain rate sensitivity coefficient, m, determined for the AZ91 (MgAl9Zn1) alloy extruded by the KOBO method at the temperature of 300 °C with the speed ε = 10^−4^ s^−1^, with the processing degree of λ = 44.4 and the die rotation frequency f = 6 Hz is 0.38. It is within the range typical for magnesium alloys with superplastic properties (0.3–0.6), which additionally allows to strengthen the thesis about superplasticity of the alloy thus produced, which by nature is not intended for plastic working.

## 4. Discussion

The article compares the structural and mechanical properties of a gravity casting to a disposable sand mold of AZ91 (MgAl9Zn1) alloy and after it has been subjected to two deformation methods: conventional extrusion at 370 °C and extrusion through a die rotating on both sides (KOBO method) at ambient temperature.

The influence of the applied deformation method on the evolution of the primary, highly segregated and therefore heterogeneous casting structure into a homogeneous, fragmented sample structure was observed. In the case of castings extruded using the conventional method, the presence of regular and equiaxed grains was observed between the highly elongated bands of the second phase. The shape and size of the grains was related to the effects of the phenomenon of dynamic recrystallization occurring during deformation at an elevated temperature. On the other hand, in the case of sand castings extruded using the KOBO method, the banding structure was revealed, correlated with the direction of extrusion. It comprised both a solid solution and a biphasic eutectic, and a pre-eutectic phase, which was also elongated in bands. A phase of the type γ-Mg_17_Al_12_ visible in the form of massive particles as a result of plastic deformation was fragmented and globularized, while locating itself in bands. The likely cause of this transformation is the cyclical change in the deformation path caused by the additional rotational movement of the tool. The shear bands running across the sample and, additionally, the local thermal factor disturbed the banding of the phase, leading to a change in its shape. A similar change of shape also concerned the discontinuous γ-phase separations, having the form of rods, bars or plates in the primary microstructure. On the other hand, a feature of castings extruded using the KOBO method were turbulences of plastic flow resulting from additional torsional movement of the die visible in the cross-sections of the sample.

On the basis of the obtained results of mechanical properties (Rm, Rp_0.2_, A) analyzed at ambient temperature, it was found that both the conventional extruded and the KOBO deformed AZ91 (MgAl9Zn1) alloy has higher strength and plasticity compared to gravity casting.

The conventionally extruded samples showed greater plasticity compared to the KOBO extruded samples. The low plasticity effect of the alloy after the KOBO process is due to the high-volume fraction of the γ phase of the Mg_17_Al_12_ type, the presence of which, from one side contributed to the increase in hardening, and on the other hand, weakens coherence of the boundaries between the solid solution. This phase could be the cause of cracks and the source of an early decohesion process during tensile testing. However, it should be emphasized that in the KOBO extrusion process, the alloy cast into a disposable sand mold had a tensile strength Rm of 437 MPa, i.e., 38% to 50% higher than conventionally extruded samples. Additionally, the change of the die rotation frequency from 3 to 6 Hz resulted in a decrease in the Rm value and an increase in the maximum deformation ε of the alloy.

An interesting feature of the sand casting extruded by the KOBO method with the processing degree of λ = 44.4 and the die rotation frequency f = 6 Hz was the elongation of 577% recorded in the static tensile test carried out at the temperature of 300 °C. Similar elongations were not recorded for the conventionally pressed sand sample. Considering the high hardening of the alloy (maximum value Rm = 437 MPa) and the presence of a finely fragmented structure with visible streaks (the average grain size in the structure of the sand casting conventionally extruded and by the KOBO method with the processing degree of λ = 44.4 measured in cross sections was, respectively, 13.46 and 2.08 μm), the superplastic behavior of foundry alloy AZ91 (MgAl9Zn1) under high temperature deformation conditions is an anomaly. It means that in the conditions of a dynamic change of the deformation path, a mechanism worked effectively to rebuild the original foundry structure with low plasticity into a structure capable of very large deformations. This is an interesting aspect in light of the subsequent forming of products of any shape and stable structure from apparently hard-deforming AZ91 (MgAl9Zn1) foundry magnesium alloys.

Due to the fact that the conditions of deformation by the KOBO method were the same as those adopted in the processes of extrusion of aluminum, zinc, titanium, and the 7075 alloy [36], the factor determining the superplastic flowability was the excess concentration of interstitial atoms generated in the conditions of cyclic change of the deformation path responsible for the visoplastic nature of the material flow.

In the literature, you can find theories about other possible causes of superplasticity in magnesium alloys deformed by SPD methods. Among them, the hypothesis related to the softening of the Mg_17_Al_12_ phase under certain thermal conditions (processes carried out at elevated temperatures) allowing for a large change in its morphology, supported by metallographic analysis, deserves attention. A special feature of this structure is the presence of fibers consisting mainly of the Mg_17_Al_12_ phase. Presumably, taking on the features of the liquid phase, it could act as a lubricating layer for the deformed matrix, contributing to the strong superplasticity of the alloy.

In another case, the main mechanisms inducing superplasticity of AZ91 (MgAl9Zn1) alloys subjected to high plastic deformation were considered to be the slip along the grain boundaries, adapted by the Coble and Nabarro–Herring creep diffusion phenomena, preventing the occurrence of structural discontinuities in the form of voids and cracks at the boundaries of the moving grains. The result of the experimental research presented in this paper were the increase in strength and plasticity of a typical foundry AZ91 (MgAl9Zn1) alloy as a result of plastic deformation by KOBO extrusion (using a die rotated on both sides).

Deformation by the KOBO method contributes to an increase in the mechanical parameters of AZ91 (MgAl9Zn1) alloy ingots obtained in the process of casting into disposable sand molds. The mechanism generating superplastic flow caused by the dynamic change of the load pattern makes the typically foundry AZ91 (MgAl9Zn1) alloy a material susceptible to further plastic forming processes. According to the literature data presented in [16,17,18,19,20,21,22,37,38], AZ91 (MgAl9Zn1) magnesium alloys are not only foundry materials. They were used during the implementation of conventional plastic working processes and severe plastic deformation (SPD) processes [6,7,8,9,10,16,17,18,19,20,21,22,23,24,25]. However, in the case of the Equal-Channel Angular Pressing (ECAP), or High-Pressure Torsion (HPT) methods, the obtained samples were small, which meant that they could be used only for basic laboratory tests. In contrast, the use of the KOBO method gives the possibility of obtaining full-size semi-finished products in the form of, among others, pipes, profiles, gears and flat bars. The course of many studies carried out so far on the KOBO process has made it possible to distinguish it from others as a low-temperature, effective method of deformation, requiring neither homogenization nor pre-heating of the ingots (carried out at room temperature) under the influence of much lower pressures than previously used deformation processes.

## 5. Conclusions

The application of the KOBO method enables plastic deformation of products made of foundry alloy AZ91 (MgAl9Zn1) without homogenization and initial heating of the charge, i.e., at room temperature. The KOBO method, based on extrusion through a die rotated on both sides, allows us to drastically increase the mechanical properties (Rm, Rp_0.2_, A) of the AZ91 (MgAl9Zn1) alloy in relation to the properties of sand casting. The results of the static tensile test carried out at the temperature of 300 °C and 350 °C proved that the sand casting samples squeezed under the conditions of deformation path change (KOBO method) acquired the features of superplastic materials with elongation reaching even 577%. The evolution of the structure of the cast ingots, which were extruded by the KOBO method, included grain refinement, the banding effect consisting of the elongation of individual structural elements such as a solid solution, the γ phase of the Mg_17_Al_12_, eutectic and pre-eutectic, correlated with the extrusion direction, and included characteristic flow swirls resulting from cyclic twisting extruded alloy.

KOBO extrusion induced significant changes in the morphology of the γ phase Mg_17_Al_12_ in the AZ91 (MgAl9Zn1) alloy in the form of its fragmentation, location in bands and globularization.

## Figures and Tables

**Table 1:** Chemical composition of AZ91 (MgAl9Zn1) alloy and the values determined in accordance with PN-EN 1753: 2020-01 [59].

Chemical Composition	The Content of Elements in wt.%								
	Al	Zn	Mn	Si	Fe	Cu	Ni	Ti	Mg
AZ91 alloy	8.5	0.75	0.13	0.02	0.02	0.01	0.002	0.025	Balance
Values according to PN-EN1753: 2020-01 [59]	8.5 ÷ 9.5	0.3 ÷ 1.0	<0.15	<0.30	<0.03	<0.025	<0.001	–	Balance

**Table 2:** Microanalysis of the chemical composition.

EDS Spot No.	Element (wt.%)		
	Mg	Al	Zn
1	58.53	36.37	5.10
2	91.50	8.50	-
3	58.25	35.50	6.24
4	90.93	9.07	-
5	80.66	16.32	-
6	83.51	13.50	-

**Table 3:** Tensile strength (Rm), yield point (Rp_0.2_) and elongation (A) of the sand casting made of AZ91 (MgAl9Zn1) magnesium alloy and after the extrusion process by the conventional method (λ = 45) and the KOBO method (die rotation frequency f = 3 and 6 Hz, λ = 44.4). Measurements performed at ambient temperature. The measurement uncertainty did not exceed 10%.

Sampling Area	Sand Cast		KOBO Extrusion, 3 Hz			KOBO Extrusion, 6 Hz			Conventional Extrusion	
	Rm (MPa)	A (%)	Rm (MPa)	Rp_0,2_ (MPa)	A (%)	Rm (MPa)	Rp_0,2_ (MPa)	A (%)	Rm (MPa)	A (%)
The beginning of the extruded wire	125	1.6	437	371	1.8	300	227	6.3	316	22
The middle part of the extruded wire			317	248	5.8	291	203	7	316	22
End of extruded wire			330	237	8.8	300	204	7.4	316	22

## Data Availability

Data sharing not applicable.
